# Methodology for the Evaluation of Varietal Resistance to *Haplaxius crudus*, Vector of the Causal Agent of Lethal Wilt in Oil Palm in Colombia

**DOI:** 10.3390/insects16020197

**Published:** 2025-02-11

**Authors:** Ivette Johana Beltrán-Aldana, Gladys Alejandra Romero-Guerrero, Eloina Mesa-Fuquen, Anuar Morales-Rodriguez

**Affiliations:** 1 Pest and Disease Program, Cenipalma, Bogotá D.C. 111211, Colombia; amorales@cenipalma.org; 2 Biology and Plant Breeding Program, Cenipalma, Bogotá D.C. 111211, Colombia; gromero@cenipalma.org; 3 Validation Unit, Cenipalma, Bogotá D.C. 111211, Colombia; emesa@cenipalma.org

**Keywords:** oil palm diversity, antixenosis, antibiosis, insect survival

## Abstract

Lethal Wilt is a limiting disease for oil palm cultivation in the eastern and central zones of Colombia. In the eastern zone, it caused the eradication of approximately 8700 ha of oil palm between 2010 and 2022, with economic losses of more than 185 million dollars. Studies conducted by Cenipalma reported that the pathogen causing this disease is *Candidatus* Liberibacter, which is possibly transmitted by *Haplaxius crudus* (Van Duzee). The adults feed on the foliage of the palms and move between them, spreading the pathogen in the plantation. A strategy to contribute to the management of LW is establishing cultivars resistant to the insect vector; however, no resistant cultivars or sources of resistance have been identified in the country’s commercial cultivars or germplasm collections. Therefore, this work aimed to design and validate a methodology to characterize the oil palm genotypes *Elaeis guineensis* and *Elaeis oleifera* and interspecific OxG hybrids against adults of *H. crudus*, evaluating resistance through antixenosis and antibiosis to identify genotypes with possible sources of resistance. An arena with leaflets of the different genotypes in free-choice tests was used to assess antixenosis. For antibiosis, entomological sleeves were installed on the palm leaves, which were infested with adults of *H. crudus* from a breeding unit. The results of antixenosis and antibiosis in both the first phase (design) and the second phase (validation) indicated greater preference and survival for the genotypes of *E. guineensis* and lower preference and survival for the interspecific hybrids and *E. oleifera*. In the genotype *E. guineensis*, the average mortality was reached after 30 days, while in *E. oleifera* and the hybrids, it occurred between the third and fourth days. The results of this research provide a reproducible methodology for the evaluation of oil palm germplasms against *H. crudus* and sucking insects for the selection of sources of resistance for incorporation into breeding programs.

## Simple Summary

Lethal Wilt (LW), caused by *Candidatus* Liberibacter and transmitted by *Haplaxius crudus*, is a disease that causes significant economic losses in palm oil plantations in Colombia, and no resistant commercial cultivars have been identified to date. This study developed and validated a methodology to evaluate the resistance of oil palm genotypes (*Elaeis guineensis*, *Elaeis oleifera*, and OxG hybrids) against the vector *Haplaxius crudus* through the mechanisms of antixenosis (feeding preference) and antibiosis (insect survival). The results indicated that *E. guineensis* is more susceptible, while *E. oleifera* and OxG hybrids showed greater resistance to the insect. The proposed methodology is a useful tool for selecting sources of resistance in breeding programs.

## 1. Introduction

Oil palm cultivation occurs in low tropical areas worldwide. Asia is the leading fruit producer at 92%, and Latin America contributes 5.4% of the world’s production, with Colombia being the main producer in the region at 1.7% [1]. The crop develops optimally at temperatures ranging from 25 °C to 38 °C, with a lower limit where plant growth is not inhibited and an upper limit for efficient photosynthesis [2]. The environmental and soil conditions for the crop fluctuate [3], favoring the adaptation of pathogens and pest insects and thus resulting in increasing phytosanitary limitations for the crop both globally [4] and regionally [5].

Lethal Wilt (LW) and Bud Rot (PC) are limiting diseases of oil palm. LW was reported in Colombia in Bajo Upía (Casanare) in 1994 [6]. In the eastern palm zone, LW caused the eradication of approximately 8700 ha of oil palm between 2010 and 2022, with economic losses of more than 185 million dollars due to the loss of productive units and management costs [7]. The disease affects the palm’s vascular system, progressively causing chlorosis in leaflets, leaf wilting, inflorescence, bunches, and roots until finally causing death.

Recent studies conducted by Cenipalma identified the pathogen *Candidatus* liberibacter as the causal agent of this disease [8]. Years ago, field studies revealed the participation of *Haplaxius crudus* (van Duzee) (Hemiptera: Cixiidae) in the dissemination of the pathogen causing Lethal Wilt, which was mainly related to the insect’s feeding habits and its relationship with the surrounding vegetation [9,10]; later, transmission studies conducted by Arango et al. [11] indicated that *H. crudus* is possibly responsible for the transmission of the disease, with an efficiency of 19%. This insect has been identified as the vector of Lethal Yellowing disease in coconut (*Cocos nucifera* L.) in Florida [12] and recently in Cuba [13]. In Colombia, its distribution is wide, occurring in three (eastern, central, and northern) of the four oil palm growing zones [14], posing an imminent threat of disease spread both in the eastern zone, which accounts for 46% of the national oil palm area, and in the central zone, where the disease was recently reported [15].

Regarding the biology of *H. crudus*, its stages are egg, nymph, and adult, it completes its entire biological cycle in grasses and sedges, and adults feed on palms. The females insert eggs at the base of the stems and under the leaf sheaths of the grasses that grow around the palms and in the pastures of the plantations [16,17]; when the nymphs emerge, they settle on the roots of the grasses to feed and remain in the nests they produce with a kind of silk that they secrete from their abdominal glands [10]. When they reach the adult stage, they fly to the palm foliage and mainly settle on the abaxial surface of the leaves, where they mate and feed on the phloem [16,18]. Thus, by feeding on diseased palms and then on healthy palms, they could transmit the pathogen causing the disease.

Studies of the population parameters of *H. crudus* under controlled breeding conditions show a high net reproductive rate, R0 = 10.96, indicating that 11 new females replace each female in the population, and with a generation time of 62.3 days, six generations would develop per year [19]. Although the climate conditions specific to each zone, as well as the variability derived from climate change, modulate the population dynamics of vector insects [20], it is evident that in the eastern zone, these conditions are appropriate, causing a significant LW impact and necessitating the development of management alternatives that contribute to reducing the economic and environmental impact of the disease.

Currently, the main management strategy for LW is directed at controlling the insect vector using insecticides, eliminating grasses where insects at immature stages are harbored, and early detection of symptomatic palms for their eradication [21]. However, in the first case, the economic and environmental impact is highly damaging. In the second case, the high eradication costs and the loss of productive units impact the financial sustainability of the crop [9].

Within integrated pest management, varietal resistance to insects is a widely used strategy, presenting advantages for crops’ economic and environmental sustainability. It occurs when a plant has chemical or structural characteristics that deter insects from feeding or ovipositing, minimizing damage compared to that in a plant that attracts the pest insect [22]. Much of the research on host plant resistance to control pest insect vectors of diseases mainly refers to the integration of antibiotic or antixenotic characteristics through conventional breeding and/or genetic engineering [23]. Antixenosis or non-preference allows plants to be incompatible with certain insects, preventing them from using the plant for oviposition, food, or shelter. This mechanism adversely affects the insects’ behavior, preventing them from parasitizing specific genotypes of their hosts. At the same time, antibiosis is the mechanism by which insects feeding on plants suffer physiological effects (temporary or permanent) and can manifest in the early death of insects, reduced growth rates, defects in pupae or adults, low energy reserves for processes such as hibernation, fecundity, and fertility, or behavioral changes [24,25].

Multiple studies have been conducted in different crops to determine the resistance by antixenosis and antibiosis of different cultivars to specific insect vectors of pathogens such as *Diaphorina citri* Kuwayama in citrus [26], *Bactericera cockerelli* (Šulc) in solanaceous [27], *Scaphoideus titanus* Ball in vine [28], *Sipha maydis* Passerini in wheat [29], *Tagosodes orizicolus* (Muir) in rice [30], *Bemisia tabaci* (Gennadius) in beans, *Sogatella furcifera* (Horváth) in rice [31], and *Myzus persicae* (Sulzer) in eggplant [32], among others. However, to our knowledge, there are no reports of evaluations of genotype resistance to hemipteran insect vectors of diseases in oil palms.

The objective of this research was to develop and validate a methodology under controlled and field conditions to evaluate the mechanisms of antixenosis and antibiosis in different oil palm cultivars (*E. guineensis*, *E. oleifera*, and interspecific OxG hybrids) for the future evaluation and identification of sources of resistance to *H. crudus* and their incorporation into breeding programs.

## 2. Materials and Methods

### 2.1. Rearing of Haplaxius crudus

Individuals of *H. crudus* were obtained from a colony established under semi-controlled conditions at the Experimental Field Palmar de Las Corocoras (CEPC) of the Corporación Centro de Investigación en Palma de Aceite in Paratebueno, Cundinamarca, Colombia, which is at an altitude of 227 m.a.s.l., latitude 4°22′04″ N, and longitude 73°10′16″ W and has an average annual precipitation of 2454 mm. These insects were originally collected locally in oil palm plantations under Resolution 02907 of 2022, which grants the Framework Permit for Collecting Specimens of Wild Species of Biological Diversity for Non-Commercial Scientific Research. These insects were reared according to the methodology of Beltrán–Aldana et al. [33] at 25.7 ± 3.4 °C, 85 ± 13% relative humidity, and a photoperiod of 12 h.

### 2.2. Plant Material

For the development of the methodology, commercial cultivars of oil palm of *E. guineensis* and interspecific OxG hybrids were evaluated, and coconut (*C. nucifera*) was used as a susceptible control. These cultivars were defined considering that they are planted in the different palm zones of the country. Based on the behavioral results of the insect vector in the commercial cultivars, in a second phase of evaluation and validation, the investigation was expanded to a set of experimental genotypes from the site’s breeding program, including the species *E. oleifera*, which is the other parent of the interspecific hybrid (Table 1). All commercial cultivars and experimental genotypes are established in research plots at the CEPC.

For the antixenosis evaluations, healthy leaflets of the corresponding leaf level were used, which were collected in the morning, transported to the laboratory with the rachises submerged in a container with water, and then kept there during the preparation of the bioassay. These leaflets were cut 20 cm long, and the rachises were covered with cotton dampened with water to keep them turgid for insect feeding.

### 2.3. Assessing Antixenosis Mechanism

**Figure 1:**
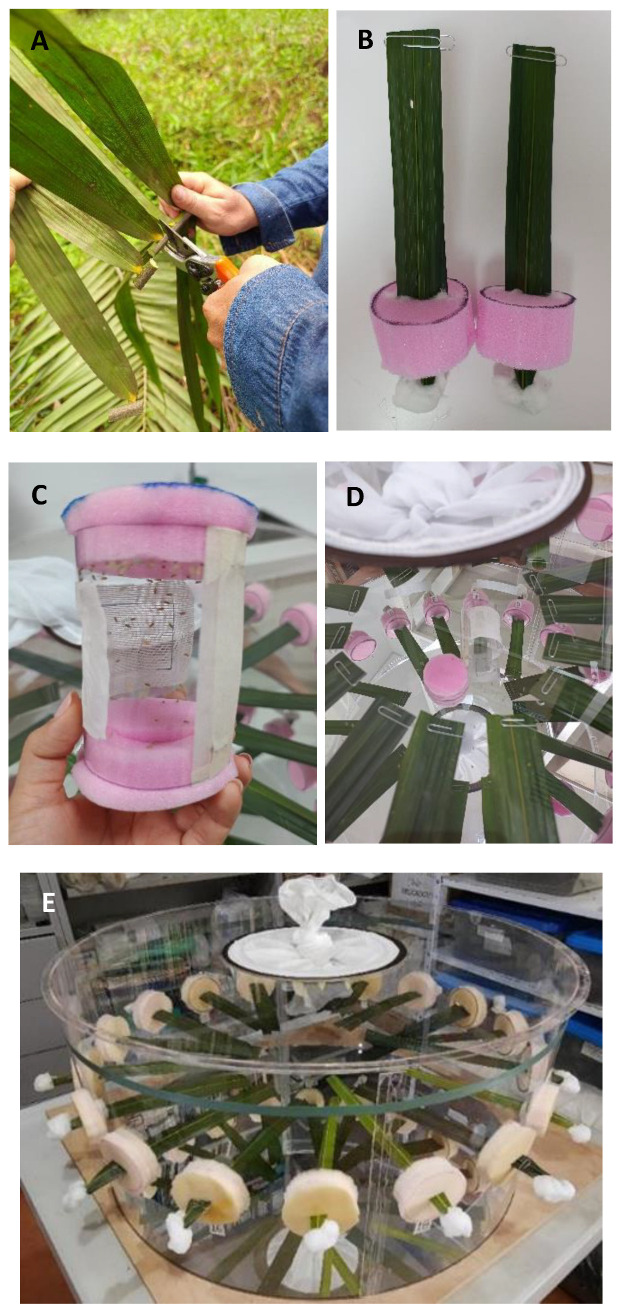
Details of the methodology used to evaluate antixenosis in free-choice tests of oil palm genotypes by *Haplaxius crudus*. (**A**) Cutting of leaflets in the field; (**B**) preparation of the leaflets to introduce them into the acrylic chamber; (**C**) cylinder with adults; (**D**) release of the adults inside the chamber; (**E**) the acrylic chamber prepared for evaluations.

Six treatments were initially included to evaluate the preference of *H. crudus* for oil palm genotypes, including five oil palm cultivars and one coconut (*C. nucifera*) cultivar as a reference control (phase 1), with a completely randomized block design with ten replications. For this, a circular acrylic chamber measuring 30 cm high × 70 cm in diameter was constructed, adapting the methodology described by Saldúa and Castro [29]. In the side wall, 18 evenly distributed holes were created, and three leaflets of each treatment were introduced; a mirror was placed at the base of the chamber to facilitate counting the adult insects perched on the underside of the leaflets, and a tulle fabric sleeve was placed at the top to introduce the *H. crudus* insects and allow air circulation (Figure 1). For the second phase, the same circular chamber was used, and 27 treatments were evaluated, including nine genotypes (three *E. guineensis* genotypes, three interspecific hybrids, and three *E. oleifera* genotypes) and three leaf levels (leaf levels 9, 17, and 25 or high, medium, and low, respectively), which were divided into three evaluation groups. In a 3 × 3 factorial structure (3 genetic origins × 3 leaf levels), the experimental unit consisted of a leaflet corresponding to each genetic origin and leaf level. In the center of the chamber, 90 unsexed adults of *H. crudus* aged 24 h old were placed. This number of insects was defined in previous evaluations where the responses of 18, 54, and 90 adults per observation chamber were monitored (Supplementary Data). After 24 h, the number of adults perched on leaflets of each genotype was counted. The antixenosis tests were conducted under laboratory conditions with a temperature of 25.6 °C ± 0.9 °C and a relative humidity of 74.8 ± 7.6%.

### 2.4. Assessing Antibiosis Mechanism

**Figure 2:**
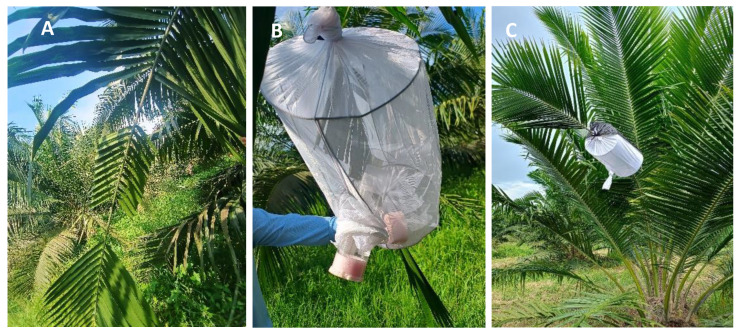
Installation of entomological sleeves for antibiosis tests. (**A**) Cutting of leaflets; (**B**) installation of the sleeve and release of the adults of *H. crudus*; (**C**) entomological sleeve located at the middle leaf level (leaf 17).

A completely randomized block design with three replications was used to evaluate the survival of *H. crudus* adults in phase 1. For these evaluations, an entomological sleeve constructed with galvanized wire (gauge 10) measuring 50 cm long by 35 cm in diameter and covered with white tulle fabric was installed (Figure 2) [34]. The experimental unit consisted of five oil palms on which three entomological sleeves were placed. In phase 2, simple random sampling of seven oil palms was conducted in plots where the nine genotypes of interest for the study were planted. Inside each cage, 25 adults of *H. crudus* aged 24 h old without prior feeding experience were placed (Figure 2B). In phase 1, the number of dead insects was recorded daily for 50 days after infestation. Based on the results, it was determined that for phase 2, the evaluation would be carried out for only 30 days. The evaluations were conducted in the field at an average temperature of 27.1 ± 4.3 °C and a relative humidity of 77.2 ± 14.0%.

### 2.5. Statistical Analysis

The data generated from the antixenosis test were analyzed to compare the effects of the different genotypes on the insect’s feeding preference; the analysis was performed using generalized linear models based on the negative binomial distribution that presented the lowest AIC (Akaike information criterion) [35] as a measure of model quality. Subsequently, the Tukey–Kramer multiple comparison test was performed when statistically significant differences in these effects were identified. For the case of antibiosis, the Kaplan–Meier method was used to calculate the probability of insect survival at any time t to then construct the survival function s(t), which was given by the relationship between the difference between the initial number insects and the number of dead insects in a period t and the total number of insects at the beginning of the test [36]. To compare the estimated curves, the hypothesis of no difference in the survival of *H. crudus* between the genotypes under study was proposed, and the log-rank test was used based on the ꭕ2 statistics. The Tukey–Kramer test was used to make pairwise comparisons based on Tukey’s studentized range test, as described in Kramer [37]. The analyses were performed with SAS 9.4 statistical software [38].

## 3. Results

### 3.1. Antixenosis to Haplaxius crudus in Oil Palm Genotypes

**Figure 3:**
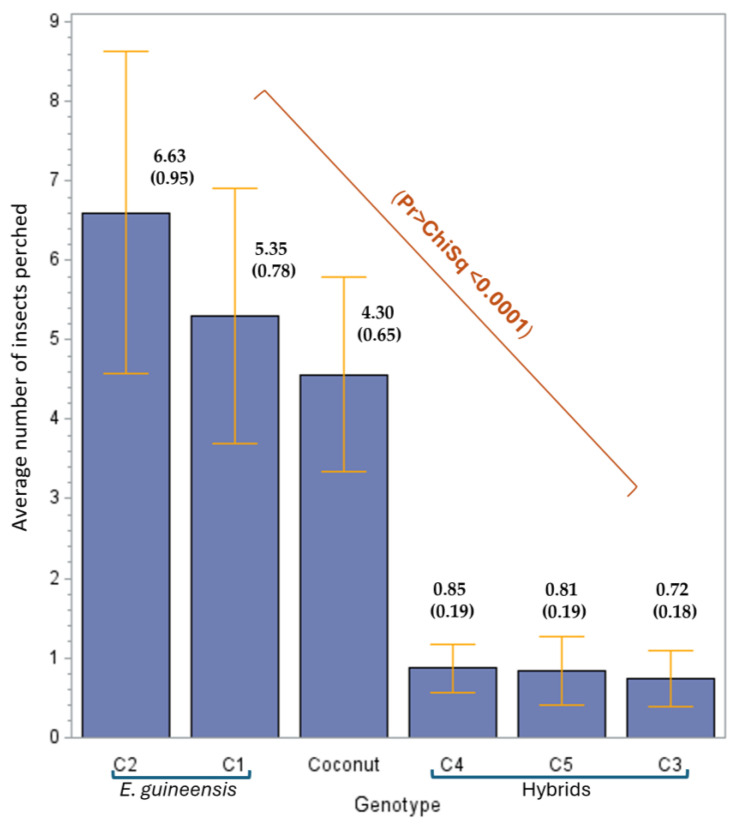
The average (standard error of the mean) number of *Haplaxius crudus* individuals perched on five commercial oil palm cultivars and one coconut cultivar based on a free-choice host test through antixenosis. C2, C1: *Elaeis guineensis* cultivars; C4, C5, and C3: commercial interspecific OxG hybrids.

In the first evaluation phase, significant statistical differences were observed in the number of *H. crudus* adults perched on the leaflets of the palm genotypes (*p* < 0.0001) at 24 h of infestation. The multiple comparison test (Tukey) showed that the genotypes could be distributed into the following two groups: the *E. guineensis* cultivars (C2, C1) and coconut, which were characterized by their higher preference for the insect, while the hybrid cultivars (C3, C4, and C5) showed a lower preference (Figure 3).

**Figure 4:**
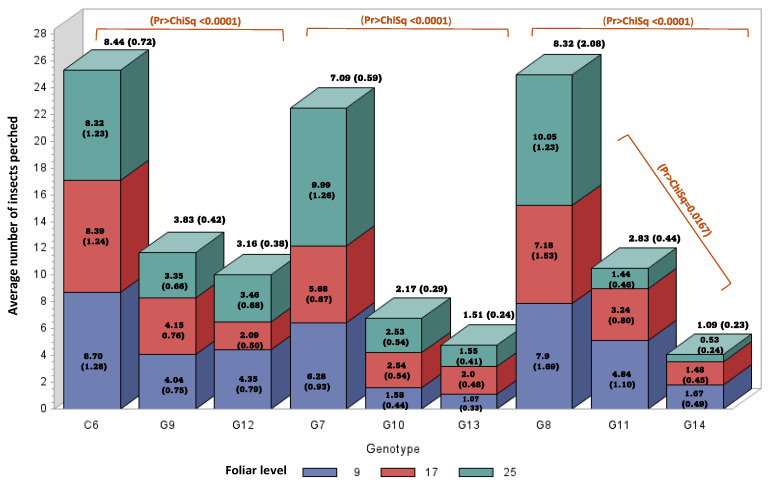
The average (standard error of the mean) number of *Haplaxius crudus* individuals perched on nine oil palm genotypes and three foliar levels based on a free-choice host test through antixenosis. C6, G7, and G8 *Elaeis guineensis* genotypes; G9, G10, and G11 interspecific OxG hybrids; G12, G13, and G14 *Elaeis oleifera* genotypes. Different colors represent leaf levels.

The results of the second phase in a larger group of genotypes, including genotypes of *E. oleifera*, showed significant statistical differences (*p* < 0.0001) in each evaluated group. The *E. guineensis* genotypes in all evaluation groups showed the highest preference (C6, G7, and G8), differing from the interspecific hybrid genotypes (G9, G10, and G11) and *E. oleifera* (G12, G13, and G14), which did not show significant differences in the first and second evaluation groups, while in the third group, there were differences for all origins (Table 1). Regarding the leaf levels, no differences were observed between them in the first two groups. In the case of the third group, an interaction effect was observed between the genotypes and the leaf level (*p* < 0.0167), as evidenced by the *E. guineensis* genotype (G8) in which leaf level 25 recorded the highest average number of insects perched, while in the interspecific hybrid and *E. oleifera* genotypes (G11 and G14), leaf level 25 presented the lowest average numbers of insects perched. However, the statistical difference between *E. guineensis* and the other two genotypes remained, and the interaction effect demonstrated magnitude but not direction (Figure 4).

### 3.2. Antibiosis to Haplaxius crudus in Oil Palm Genotypes

**Figure 5:**
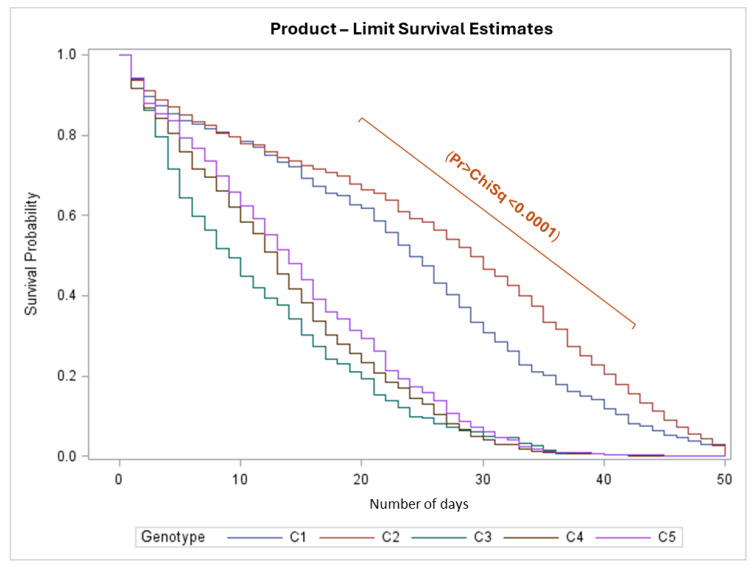
Survival curves of *Haplaxius crudus* in commercial oil palm cultivars *Elaeis guineensis* (C1 and C2) and interspecific OxG hybrids (C3, C4, and C5).

In phase 1, significant differences (*p* < 0.0001) were observed in the survival curves. The multiple comparison test separated the *E. guineensis* cultivars from the interspecific hybrids (Figure 5). The cultivars C3, C4, and C5 (interspecific OxG hybrids) reached 50% mortality of *H. crudus* between 10 and 15 days after infestation. In contrast, in the cultivars C1 and C2, 50% mortality occurred between 25 and 30 days.

**Figure 6:**
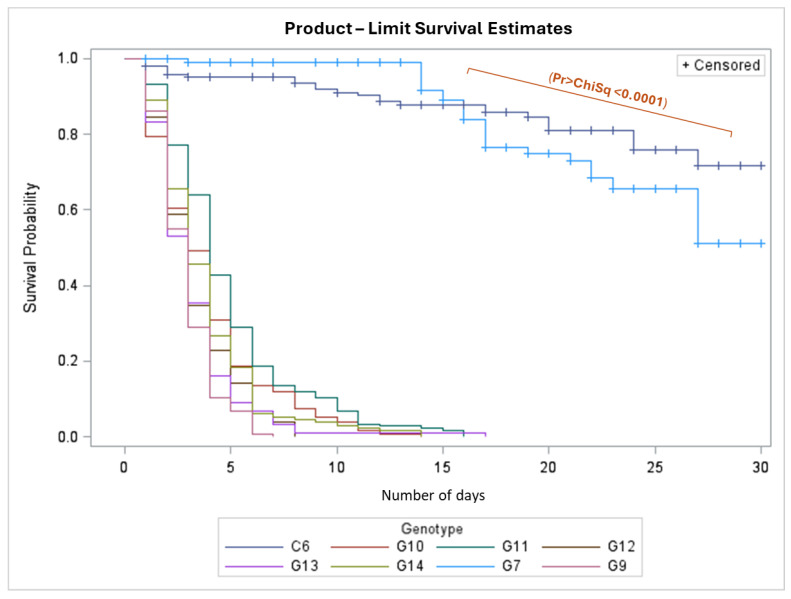
Survival curves of *Haplaxius crudus* in different oil palm genotypes determined using the Kaplan–Meier method.

In validating the methodology (phase 2), the probability of survival of *H. crudus* showed significant differences for the evaluated genotypes. Once the survival curves depicting s(t) were constructed, they were compared with the log-rank test, which showed significant statistical differences (*p* < 0.0001). The results were consistent with those from the first phase. For the *E. guineensis* genotypes, survival was higher, with mortality not reaching 50% in the infested population at 30 days, while in the interspecific OxG hybrids and *E. oleifera* genotypes, 50% mortality was reached between days 3 and 4 after infestation (Figure 6). Significant differences were found between *E. guineensis* (C6 and G7) and *E. oleifera* (G12, G13, and G14), between *E. guineensis* and the interspecific OxG hybrids (G9, G10, and G11), between *E. oleifera* G13 and interspecific hybrid G11, and within the interspecific hybrids G9 and G11. The genotype G8 was not considered in the analysis because not all insects within each experimental unit died, and since the experimental unit consisted of several insects, it was censored.

## 4. Discussion

The results of antixenosis and antibiosis to *H. crudus* in both the first and second phases indicated greater preference and survival for the genotypes of *E. guineensis* and lower preference and survival for the interspecific hybrids and *E. oleifera*. It is essential to highlight that these results were consistent between the two tests, noting that *E. guineensis* and interspecific OxG hybrids were included in the first phase. In addition to *E. guineensis* and interspecific OxG hybrids, *E. oleifera* genotypes were included in the second phase. The American palm oil *E. oleifera* species is a genetic resource that gave rise to the interspecific OxG hybrids and is characterized by its resistance to Bud Rot disease compared to commercial *E. guineensis* cultivars, which are highly susceptible in focal areas [39]. *Elaeis oleifera* genotypes have also been evaluated for their behavior against the leaf miner *Coelaenomenodera lameensis* Berti (Coleoptera: Chrysomelidae), indicating that the American species is more resistant than *E. guineensis* and presents morphological characteristics such as a thicker epidermis and cuticle [40].

The methods for evaluating antixenosis are traditional and have allowed germplasm studies in different crops against different pest insects considering the damage caused by feeding [41,42,43]. While probing techniques were not tested [44,45], further investigation related to histological characterization and volatile and non-volatile metabolite determination is in progress to try to determine resistance traits that might be involved in insect preference and non-preference for these cultivars. Additionally, variables associated with oviposition were not measured, as in the insect’s biological cycle, it uses grasses for oviposition, and only the adult feeds on the oil palm [16,18]. This feeding variable was evaluated in the field through the survival (antibiosis) of adults on oil palm, representing a fundamental function that influences the rates of oviposition, which starts between the 4th and 6th day of age (unpublished data); females that do not survive this period have a lower probability of leaving offspring, which would be the case considering the mortality times found for the interspecific OxG hybrids and *E. oleifera* genotypes (Figure 6).

Among the plant mechanisms for insect resistance responses, morphological traits such as a thick epidermis, waxy cuticles, spines, and higher trichome density [22,46,47] have been found to act as physical barriers for adhesion, feeding, and oviposition by insect pests. Traits like these should be studied in the *E. oleifera* species and the interspecific OxG hybrids to determine their relationship in antixenosis and antibiosis to *H. crudus*. Variation in secondary metabolites such as phenolic compounds and terpenes has been observed in citrus resistant to *D. citri* [26,48]. In oil palm, peaks of polyphenols were observed in leaf extracts of *E. oleifera* palms that resulted in higher mortality for *C. lameensis* larvae compared to extracts of *E. guineensis* and interspecific OxG hybrids [49,50].

Several studies mention that structural and chemical traits that integrate plant resistance to vector insects hinder the transmission of diseases, mainly those of viral origin [51,52], and the development of genetic resistance against vector insects is often sought to manage viral diseases [53]. In the case of aphids, knowledge of plant defense mechanisms against these insects (antibiosis and antixenosis) has led to the identification of resistance genes and signaling mechanisms that contribute to defense and plant defense elicitors derived from aphids [54].

The results of this research provide a reproducible methodology for evaluating oil palm germplasms against *H. crudus* and sucking insects for the selection of resistance sources for incorporation into breeding programs aimed at finding cultivars with resistance characteristics to vector insects as a strategy for integrated management [55]. The results obtained by comparing the interspecific OxG hybrids and genotypes of the two progenitor species of this hybrid indicate that the interspecific hybrid and *E. oleifera* genotypes are less attractive for insect feeding, thus presenting resistance to the insect, and there is a high probability that the hybrid inherits this characteristic from *E. oleifera*, showing similar responses. It is also possible to consider that there is a partial dominance genetic effect conferred by *E. oleifera* for these characteristics; however, it is necessary to conduct further research to determine the morphophysiological and genetic mechanisms that determine this differentiated response.

## Figures and Tables

**Table 1:** The commercial cultivars and experimental genotypes of oil palm (*E. guineensis*, OxG hybrids, and *E. oleifera*) evaluated. The experimental genotypes (letter G) in the table are part of the Cenipalma oil palm breeding program, which are defined by their productive potential (hybrids) and promising progenitors (*E. oleifera*). Commercial genotypes are denoted with the letter C.

**Phase 1**			
**Cultivar/Genotype**	**Name/Code**	**Genetic Cross**	**Genetic Origin**
C1	IRHO 1001	Deli × La Mé	*Elaeis guineensis*
C2	Unipalma	Mo.Co × C.Mix	*Elaeis guineensis*
C3	Amazon 17	Manaos × Compacta	Interspecific Hybrid
C4	Unipalma 2658	Brazil × (Avros × Djongo)	Interspecific Hybrid
C5	Cabaña OxG 0056	Coarí × La Mé	Interspecific Hybrid
**Phase 2**			
**Cultivar/Genotype**	**Name/Code**	**Genetic Cross**	**Genetic Origin**
C6	IRHO 1001	Deli × La Mé	*Elaeis guineensis*
G7	Experimental 5-1-4-5	Angola × Tester	*Elaeis guineensis*
G8	Experimental 5-2-2-1	Angola × Tester	*Elaeis guineensis*
G9	Experimental 01	OxG	Interspecific Hybrid
G10	Experimental 26	OxG	Interspecific Hybrid
G11	Experimental 64	OxG	Interspecific Hybrid
G12	Experimental 57	OxO	*Elaeis oleifera*
G13	Experimental 87	OxO	*Elaeis oleifera*
G14	Experimental 56	OxO	*Elaeis oleifera*

## Data Availability

Data supporting this research are available from the corresponding authors upon request.

## Supplementary Materials

The following supporting information can be downloaded at: www.mdpi.com/article/10.3390/insects16020197/s1, Table S1: Percentage of preference according to the number of insects per chamber.
