# Supplementary material for: Methodology for the Evaluation of Varietal Resistance to Haplaxius crudus, Vector of the Causal Agent of Lethal Wilt in Oil Palm in Colombia
Source: Insects. 2025 Feb 11;16(2):197. doi: 10.3390/insects16020197 (PMC11856791; doi:10.3390/insects16020197)
Supplement: Supplementary file 1 [file insects-16-00197-s001.zip › insects-3405895-supplementary.pdf]

Table S1. Percentage of preference according to the number of insects per chamber.

Cultivars	% Preference/number of insects per chamber (Mean ± SD)			
	18 insects	54 insects	90 insects	90 insects (Rep)
**Coconut**	13,9 ± 12,4	8,3 ± 6,8	14,6 ± 5,6	15,2 ± 9,4
**C1**	7,8 ± 8,8	8,0 ± 5,5	13,0 ± 6,5	17,7 ± 6,9
**C2**	6,1 ± 6,7	8,5 ± 6,5	12,4 ± 9,4	22,0 ± 12,7
**C3**	2,8 ± 5,4	4,8 ± 3,5	4,0 ± 3,5	2,9 ± 2,0
**C4**	3,9 ± 4,6	3,9 ± 4,7	3,9 ± 3,2	2,8 ± 2,9
**C5**	7,2 ± 5,9	5,7 ± 4,0	2,7 ± 2,3	2,4 ± 1,9
